# NetB, a Pore-Forming Toxin from Necrotic Enteritis Strains of *Clostridium perfringens*

**DOI:** 10.3390/toxins2071913

**Published:** 2010-07-23

**Authors:** Anthony L. Keyburn, Trudi L. Bannam, Robert J. Moore, Julian I. Rood

**Affiliations:** 1 CSIRO Livestock Industries, Australian Animal Health Laboratory, Geelong, Victoria 3220, Australia; Email: Anthony.Keyburn@csiro.au (A.K.); 2 Australian Research Council Centre of Excellence in Structural and Functional Microbial Genomics, Department of Microbiology, Monash University, Victoria 3800, Australia; Email: trudi.bannam@monash.edu (T.L.B.); Rob.Moore@csiro.au (R.J.M.)

**Keywords:** NetB, necrotic enteritis, *Clostridium perfringens*, pore-forming toxin

## Abstract

The *Clostridium perfringens* necrotic enteritis B-like toxin (NetB) is a recently discovered member of the β-barrel pore-forming toxin family and is produced by a subset of avian *C. perfringens* type A strains. NetB is cytotoxic for avian cells and is associated with avian necrotic enteritis. This review examines the current state of knowledge of NetB: its role in pathogenesis, its distribution and expression in *C. perfringens* and its vaccine potential.

## 1. Introduction

*C. perfringens* is a Gram-positive anaerobe and is ubiquitous in the environment, being found in soil, in decaying organic matter, and as a member of the normal intestinal flora of many humans and animals [1]. Specific *C. perfringens* toxin types are associated with particular human and animal diseases, indicating that variations in toxin production influence the virulence properties of *C. perfringens* isolates [2,3,4]. *C. perfringens* strains are typed by the production of four major toxins (α, β, ε, ι) [3]. Type A strains are the most widespread and are commonly found in the intestines of warm blooded animals and in the environment. Type A strains cause gas gangrene in humans and enteric diseases in humans and animals [5,6,7,8]. Type C strains cause mucosal necrosis of the small intestine in humans and pigs, while type B and C strains cause enteric disease in small ruminants [5]. 

**Figure 1:**
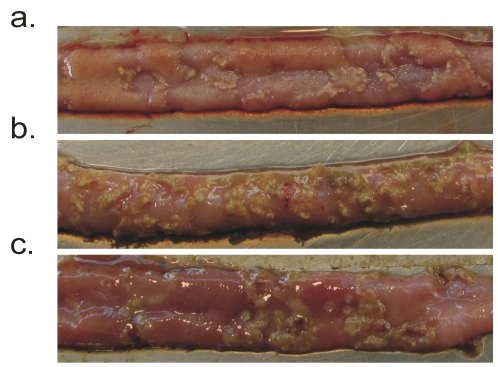
Gross pathology of the small intestine of infected birds. (**a**–**c**) are three examples of necrotic enteritis lesions in the small intestines of broiler chickens challenged with *C. perfringens*.

Necrotic enteritis in poultry is a global problem and it has been estimated that the disease costs the international poultry industry in excess of $US 2 billion per year in production losses and control measures [9,10,11]. Avian necrotic enteritis is an enteric disease that is characterized by necrotic lesions in the small intestinal mucosa [12]. The disease can be divided into two forms, clinical and subclinical. The clinical signs of acute necrotic enteritis can be seen from about two weeks of age and include marked depression, dehydration, apathy, diarrhea, ruffled feathers, and decreased feed consumption [13,14,15,16]. Birds showing clinical signs normally die within a few hours, with mortality rates reaching up to 1% per day [13]. The diagnosis of subclinical necrotic enteritis is based on a decrease in the feed conversion ratio and the detection of gross necrotic lesions in the small intestinal mucosa (Figure 1) followed by bacteriologic analysis and genotyping of isolates [12]. Macroscopic lesions can be seen in the small intestine and can sometimes be found in other organs, such as the liver, kidney and cecum [17]. In an affected bird, the small intestine can become enlarged due to gas accumulation and the wall of the intestine can become thin and delicate [18]. Subclinical necrotic enteritis is observed at varying ages of birds, but most commonly it is first detected in birds at 21 to 23 days of age [9]. The subclinical form of the disease is not only of significance to the animals’ welfare, but also to the producer through lost productivity and the added cost of disease intervention. 

All *C. perfringens* strains produce α-toxin, a major extracellular toxin that has been shown to be essential for human myonecrosis [19]. It was previously thought [14,20,21] that α-toxin was the major toxin involved in necrotic enteritis, but we have shown that an α-toxin null mutant is still virulent in a chicken necrotic enteritis disease model [22]. This result led us to postulate that virulent necrotic enteritis strains produce other toxins that are responsible for the pathogenesis of disease. Recently, we identified a new toxin, NetB, in avian *C. perfringens* type A strains [23]. NetB is a key virulence factor in *C. perfringens* strains that cause avian necrotic enteritis. Here we review our current knowledge of the biology, distribution, regulation, role in disease, and potential vaccine applications of this new toxin.

## 2. Identification and Analysis of NetB

**Figure 2:**
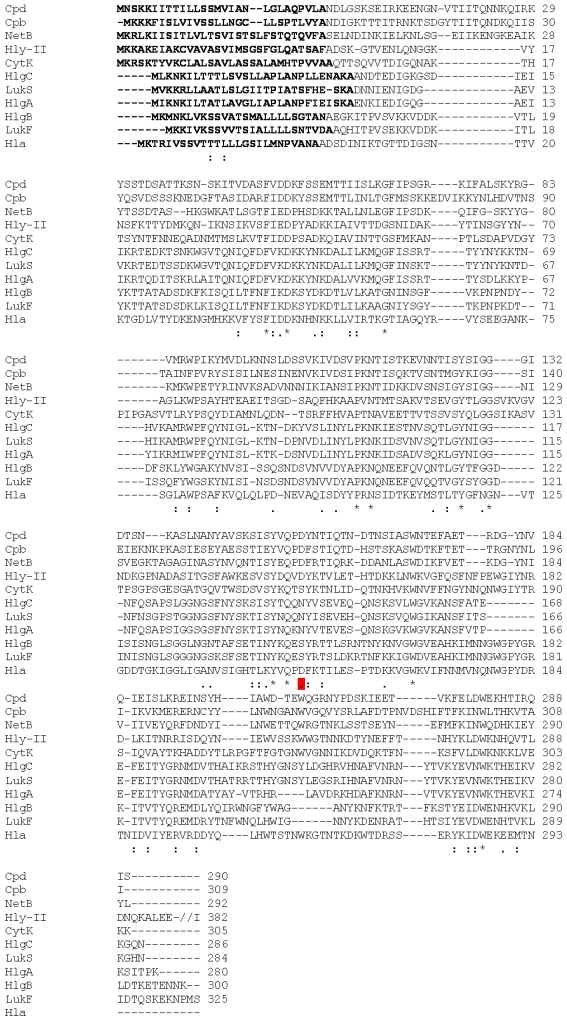
ClustalW alignment of NetB. ClustalW alignment of the toxins *C. perfringens* NetB (EU143239), *C. perfringens* β-toxin (Cpb, AAA23284.1), *C. perfringens* δ-toxin (Cpd, ACF93710.1), *B. cereus* hemolysin II (Hly-II, NP_833256.1), cytotoxin K (CytK, CAC08441.1) and *S. aureus* α-toxin (Hla, NP_371687.1), γ-toxin A (HlgA, P0A074.1), B (HlgB, P0A077.1), C (HlgC, Q07227.1), Leukocidin S (LukS, NP_058465.1), F (LukF, NP_058466.1). Identical residues (*), conservative amino acid substitutions (:) and semi-conservative amino acid substitutions (.) are shown below the aligned sequences. Residues highlighted in bold represent the known or predicted signal peptide sequence of each protein and residue numbers begin following the signal peptide sequence. Red boxes indicate those residues that are known to be involved in the activity of at least one of these proteins, as discussed in the text. To compact the figure the Hly-II sequence was shortened.

NetB was discovered in an Australian strain of *C. perfringens* type A that was isolated from a chicken with necrotic enteritis [23]. Earlier work, aimed at defining the role of α-toxin, had indicated that secreted products from *C. perfringens* were able to cause lesions typical of necrotic enteritis in chickens [20]. Based on these results, and our finding that α-toxin was not required for disease, we tested culture supernatant from our α-toxin mutant against various mammalian and chicken cell lines to determine whether any novel cytotoxic factors could be identified. One cell line, LMH (ATCC CRL-2117), was found to be sensitive to an unknown protein in the supernatant. Subsequently, LMH cytotoxicity was used to follow this toxic activity during the purification process. The *N*-terminal sequence of the purified protein was determined and used to search the genome sequence of the parent *C. perfringens* strain, EHE-NE18. A novel toxin gene that encoded a 323 amino acid protein, including a 30 amino acid secretion signal sequence, was identified. Since this protein has similarity to *C. perfringens* β-toxin (38% identity) it was designated necrotic enteritis toxin, B-like (NetB) [23]. In addition to β-toxin, NetB has sequence identity to *C. perfringens* δ-toxin (40% identity), *Staphylococcus aureus* α-hemolysin (30% identity), the A, B and C components of *S. aureus* γ-toxin (25%, 22% and 23% identity, respectively), the S and F components of *S. aureus* leukocidin (25% and 22% identity, respectively) and to *Bacillus cereus* cytotoxin K (27% identity). Alignment of these sequences (Figure 2) reveals that only 14 amino acids are identical in these proteins and that 50 residues are conservatively substituted. The areas of similarity are dispersed throughout the toxin, with the weakest similarity in the *N*-terminal region. 

At least 35% of the known protein toxins produced by bacteria belong to the pore-forming group of membrane damaging toxins [24]. These toxins form pores that disrupt the phospholipid membrane bilayer of both human and animal cells, causing an influx of ions (*i.e.*, Na^+^, Cl^−^, Ca^2+^, *etc.*) that may lead to osmotic cell lysis. Many of these toxins have been demonstrated to contribute to the virulence of bacteria and to play a key role in the pathogenesis of human and animal infections [25,26,27,28,29,30,31]. NetB has many of the conserved residues found in pore-forming toxins of the *S. aureus* toxin family [32]. For example, the α-hemolysin, or α-toxin, from *S. aureus* is a secreted, self-assembling, pore-forming toxin [33]. It has a signal peptide of 26 amino acids and in its mature form consists of a 293 amino acid polypeptide with a calculated molecular size of 33 kDa [34,35]. β-toxin also is a member of this pore‑forming family and is a lethal extracellular toxin produced by *C. perfringens* type B and C strains [36]. β-toxin is secreted in late exponential growth phase and is extremely susceptible to proteases and to heat [37,38]. Located on a plasmid [39], the β-toxin structural gene, *cpb*, encodes a 336 amino acid polypeptide that includes a 27-amino acid signal sequence, which is cleaved to form the mature β-toxin protein (34 kDa) [40]. 

Site-directed mutagenesis has been used to characterize many amino acids critical for function in *C. perfringens* β-toxin and *S. aureus*α-toxin [41,42] and NetB contains many of these key residues including R200 (R212 from β-toxin). The corresponding residue in *S. aureus* α-toxin (R200) is important for binding and oligomerization of the protein as well as for hemolysis [43]. Mutation of the β-toxin residue Y203 (Y191 in NetB) to phenylalanine resulted in a 2.5-fold increase in the LD_50_. The corresponding residue in *S. aureus* α-toxin (Y191) is located at the predicted membrane binding surface of the protein [44]. Mutation of the β-toxin residue D167 (D156 in NetB) resulted in a complete loss of functional protein expression, suggesting that this region participates in crucial protomer-protomer interactions as well as being important for conformational rearrangements involved in multimer formation in the membrane bound form [44]. 

Since NetB had many of the residues essential for β-toxin and *S. aureus* α-toxin function we postulated that NetB was likely to be a pore-forming toxin. Subsequent studies demonstrated that NetB was found to be cytotoxic to LMH cells but not to other chicken cell lines such as DF1 and HD11 cells or the mammalian Vero cell line [23]. NetB (2.5 ng/µL) causes significant cell rounding and cell lysis in as little as 30 min. Incubation with PEG 1000 and PEG 1500 blocked these morphological changes and LDH release from the cells, indicating that NetB most likely forms plasma membrane pores with an estimated pore diameter of 1.6 to 1.8 nm [23]. This NetB pore diameter is slightly larger than the pore size predicted for β-toxin (1.36–1.6 nm) in HL 60 cells [45]. β-toxin has been shown to induce significant host cell swelling without blebbing, cell lysis, and the release of K^+^ from human leukemia cells (HL 60) [45]. Incubation of HL 60 cells with β-toxin resulted in the formation of toxin complexes of about 191 kDa (hexamer) and 228 kDa (heptamer). The 228 kDa complex was observed up to 30 min after incubation with the cells, while the 191 kDa complex remained after 60 min. 

δ-toxin is another *C. perfringens* pore-forming toxin that has sequence identity with NetB [46]. This toxin is a hemolysin released by several *C. perfringens* type C and possibly type B strains [47]. It is cytotoxic for various cell types such as rabbit macrophages, human monocytes, and blood platelets from goats, rabbits, humans and guinea pigs [46,48,49,50]. The toxin forms pores in artificial lipid bilayers similar to β-toxin, however, these toxins do not share a similar receptor since δ-toxin binds to gangliosides (G_M2_) unlike β-toxin [46]. Some *C. perfringens* type B and C strains that are involved in necrotic enteritis in various animal species, mainly piglets, and also in humans, produce δ-toxin, but no direct link between this toxin and disease has been demonstrated. 

The essential role of NetB in disease was determined by constructing a structural gene (*netB*) mutant in strain EHE-NE18 and assessing its virulence in a chicken disease model [23]. Virulence testing of an isogenic series of strains consisting of the wild type, *netB* mutant, and *netB* mutant complemented with the wild-type *netB* gene, revealed that the development of necrotic enteritis in chickens was dependent on the ability to produce functional NetB toxin. This result parallels studies on the α-toxin of *S. aureus*, which in a mouse model of *S. aureus* pneumonia has been shown to be an important virulence factor of methicillin-resistant *S. aureus* (MRSA) [51]. *S. aureus* α-toxin negative mutants were found to be 10-fold less virulent than α-toxin producing strains. Similar results have been obtained for β-toxin and it has been shown to be essential for hemorrhagic necrotizing enteritis in rabbit ileal loops [52]. In that study, mutations in the β-toxin structural gene, *cpb*, were constructed and shown to be avirulent in the ileal loop model. Complementation of a *cpb* mutant restored its ability to produce β-toxin and restored virulence in the loop model. In addition, highly purified β-toxin was able to reproduce intestinal damage that was similar to infection with the wild-type *C. perfringens* strain. Both NetB and β-toxin act in the gastrointestinal tract of animals, however, β-toxin has also been suggested to cause toxemia after absorption of the toxin from the intestines into the circulation [53,54]. However, no experiments aimed at determining if NetB enters the circulatory system of infected chickens have been reported. 

Recent studies have revealed that in several necrotic enteritis isolates the *netB* gene is part of a large potential pathogenicity locus [55]. Comparative analysis of genomic sequences from seven independent disease-causing *C. perfringens* isolates and a range of non-necrotic enteritis strains showed that the *netB* gene is located on a 42 kilobase (kb) locus (NELoc-1). In addition, Southern blots suggest that the *netB* gene is located on an approximately 85 kb plasmid. From the genomic analysis, two other smaller loci (NELoc-2 and NELoc-3) also appear to be associated with necrotic enteritis strains. The identification of these pathogenicity loci may lead to the discovery of additional virulence factors that are involved in the pathogenesis of disease. 

## 3. Strain Distribution

*C. perfringens* is commonly present within the normal flora of the gastrointestinal tract of chickens. However, under conditions that are still not fully understood, birds can develop necrotic enteritis, with disease progression correlating with the proliferation of *C. perfringens* cells in the intestine. To understand what types of *C. perfringens* isolates are involved in this changing ecological balance, several studies have examined the clonal variation of *C. perfringens* isolates. Epidemiological studies of strains from varied geographical locations have shown that healthy chickens harbor a range of genotypes, as determined by PCR, PFGE and MLST studies, while isolates from birds present in individual flocks that have acute necrotic enteritis tend to have only one genotype of *C. perfringens* [56,57,58,59,60,61,62,63,64]. In contrast to these findings, which involved acute disease outbreaks, in one study birds from a single flock that displayed mild necrotic enteritis symptoms were found to harbor a genetically varied range of isolates [64]. 

Since the discovery of NetB [23] several studies have screened for the presence of the *netB* gene within a wide variety of *C. perfringens* strains. Initial screening of a range of Australian poultry necrotic enteritis isolates found that the majority (77%) were *netB* positive [23]. In addition, *in vitro* expression analysis confirmed that all of the *netB* positive strains that were tested produced the NetB protein. Furthermore, several non-necrotic enteritis strains were analyzed and found to be *netB* negative. These *netB*-negative strains included representatives of each of the toxin types A to E, which had been isolated from cattle, sheep, pigs and humans. Subsequent studies that screened for the *netB* gene in strains of *C. perfringens* from other geographical regions showed that there is a good correlation between the isolation of *C. perfringens* from a diseased bird and the presence of the *netB* gene [59,64,65,66]. In combination, 60 to 90% of the strains isolated from birds with necrotic enteritis were found to have the *netB* gene. Chalmers *et al.* [59], studied Canadian *C. perfringens* strains, and found that the *netB* gene was detected only in isolates associated with necrotic enteritis outbreaks; it was not identified in isolates from healthy birds. Analysis of isolates from the USA showed that the *netB* gene was in 60% of necrotic enteritis strains (7/12) [65]. Examination of a single broiler flock from Sweden found that more than 90% of all isolates from necrotic enteritis-affected poultry were *netB* positive [64]. The same trend was found in an extended study of strains from three continents [66], where 70% of isolates from necrotic enteritis affected birds were *netB* positive. Together, these results suggest that the NetB toxin is necrotic enteritis specific, since the *netB* gene is strongly associated with necrotic enteritis-derived strains from poultry. Until recently, the *netB* gene had only been found in poultry related strains, however, the first non-poultry based recovery of a *netB* positive *C. perfringens* isolate has now been reported. A *netB* positive strain was isolated from liver abscesses from a cow that had died with gastrointestinal disease, although this particular *C. perfringens* strain was not considered to be the causative agent [65]. In a chicken disease model, this bovine isolate has also been shown to cause lesions characteristic of avian necrotic enteritis [67]. 

Not all *C. perfringens* strains isolated from birds that clearly displayed signs of necrotic enteritis were *netB* positive. In nearly every study, there was a minor fraction of *netB* negative isolates from diseased birds [23,59,64,65,66,68]. In one particular study [65] a *netB* negative isolate was recovered from a chicken displaying clear signs of necrotic enteritis. To show that there were no sampling errors involved, 25 isolates were recovered from different lesion areas of the same chicken; they were all *netB* negative. These results imply that although there is a clear association between production of NetB toxin and development of the pathology of necrotic enteritis, there may be other—yet to be determined—virulence factors that are produced by these *netB* negative disease-producing strains. By contrast, several *netB* negative strains isolated from lesion material have been tested for their ability to cause disease in necrotic enteritis animal models and found to induce little or no signs of disease [66,67,69]. Importantly, one *netB* negative strain, JGS4104, induced a significant level of disease (43.8%) [69], again suggesting that there may be other factors, besides NetB, that allow *C. perfringens* type A to cause tissue damage similar to necrotic enteritis. In this study the strains re-isolated from the diseased birds were analyzed by PFGE. Given the recent demonstration that the *netB* structural gene is plasmid borne [55], it is unclear if it is possible using PFGE to detect a difference between a strain that remains *netB* negative and one that has obtained the NELoc-1-containing plasmid. Clearly, more research needs to be done to test and assess the virulence of additional isolates of different origins and genotypes in the chicken disease model. 

Whilst the *netB* gene has been found mainly in association with poultry with necrotic enteritis, it has also been identified in *C. perfringens* strains from healthy birds. In one study [65], 8.8% of isolates (7/80) from normal chickens were *netB* positive. In another study, analysis of strains from a single broiler flock that contained birds affected with mild necrotic enteritis, revealed that 25% of the isolates from apparently healthy birds were *netB* positive [64]. Nowell *et al.* 2009 [70] used an enrichment process to recover 88 *C. perfringens* isolates from retail grocery chicken samples and found that the *netB* gene could be detected in 21% of these isolates. Therefore, as previously suggested, it appears that the presence of a *netB* positive strain is not sufficient to cause disease, predisposing conditions are required. Analysis of strains from various Danish broiler flocks found that 61% of isolates recovered from healthy birds were *netB* positive [68]. This was markedly different from the results obtained from previous studies on strains from healthy birds [59,65,66]. When these Danish *netB* positive isolates were tested for *in vitro* expression of the NetB protein, a marked difference in the NetB expression rates was observed [68]. The NetB protein was found in 12 out of 13 of the isolates from the necrotic enteritis diseased birds. By comparison, only four out of 14 PCR positive isolates from healthy birds produced detectable NetB protein. These results are in stark contrast to our own findings, where every strain that carries *netB* produces NetB protein. It would be interesting to test these NetB producing and NetB non-producing *netB* positive strains isolated from healthy birds for NetB expression *in vivo* and to determine if they are equally capable of disease production. 

## 4. Regulation of NetB Production and NetB Sequence Variation

Expression of the *netB* gene is regulated by VirSR, the global regulator of *C. perfringens* toxin production [71]. In the human gas gangrene-causing *C. perfringens* type A strain 13, the VirSR system is part of a complex signal transduction pathway that directly regulates the production of extracellular toxins and enzymes such as perfringolysin O and α-clostripain and regulates the expression of α-toxin, collagenase and many housekeeping genes by controlling the expression of the regulatory RNA molecule, VR-RNA [72]. In two separate necrotic enteritis derived *C. perfringens* strains, mutation of the *virR* gene greatly reduced the production of NetB toxin [71]. This effect was reversed when the *virR* mutants were complemented with the wild-type *virR* gene. VirR acts on its target genes by binding to two directly repeated sequences, or VirR boxes, that are located immediately upstream of the promoter. Similar repeats are located upstream of the putative *netB* promoter. These VirR boxes were shown to be functional in a *C. perfringens*-based bioassay and *in vitro* studies showed that purified VirR protein could bind to the *netB*-associated VirR boxes. Other workers have shown that in strain 13 the VirSR system appears to be regulated by an Agr-dependent quorum sensing system that leads to the controlled expression of specific genes in response to changes in cell density [73]. These findings suggest that in the gastrointestinal tract of infected birds the production of NetB is activated when the *C. perfringens* population reaches a threshold level as a result of predisposing factors, as reviewed previously [17,74], and nutrients start to become rate-limiting for growth. 

Any differences in necrotic enteritis disease progression could potentially be explained by sequence differences in the NetB proteins produced by different strains. To examine the variation of NetB proteins, the nucleotide sequence of the *netB* gene from a range of isolates was determined [66]. The poultry isolates examined were from various geographical locations including Australia, Belgium, Denmark and Canada. The *netB* gene and its promoter region displayed very little sequence variation; most of these strains had virtually identical nucleotide sequences in this region. Within the *netB* coding sequence there was only one amino acid change, A168T, which was observed in six strains. Biological analysis of the NetB_A168T_ derivative showed that it was as active as the wild-type NetB protein and that strains carrying the variant sequence produced similar levels and types of disease when used in infection studies. The observation that NetB is very well conserved was supported by a separate study [68], which found that most of the 27 *netB* positive isolates examined contained wild-type coding and promoter regions. The only amino acid sequence changes observed were NetB_A168T_, the variant previously reported, and a single example of a novel variant, NetB_A166V_. 

## 5. NetB and the Development of a Necrotic Enteritis Vaccine

Since NetB is essential for disease pathogenesis, and is a secreted toxin that should be readily accessible to the host immune system, it has considerable potential for vaccine development [23]. Vaccines against *C. perfringens* or its toxins have been successfully used to prevent enteric diseases in various mammalian species, including humans [75,76,77]. Vaccine development for necrotic enteritis in chickens has previously focused on α-toxin, but the experimental vaccines produced have not provided the level of vaccine efficacy seen with clostridial vaccines in other animal species, presumably because α-toxin is not essential for virulence. It has been shown that chicken flocks with high levels of maternal antibodies against α-toxin have lower mortality levels than flocks with lower titers [78]. Vaccines based on *C. perfringens* type A and C have been experimentally tested [79]. Layer hens vaccinated with these toxoids had high levels of anti-α-toxin antibodies in their progeny compared to unvaccinated hens. Both toxoid vaccines showed levels of protection against subclinical necrotic enteritis, with the type C toxoid vaccine giving higher levels of protection than the type A toxoid. α-toxin has also been delivered as a subunit vaccine and by live delivery in *Salmonella* vectors, where it has been shown to provide some protection [80,81,82]. Recently, a commercial vaccine (NETVAX®, Schering-Plough) has been developed based on a *C. perfringens* type A toxoid. However, the NetB status of the organisms used to develop this vaccine is not known. 

Other studies aimed at the development of a necrotic enteritis vaccine have been reported. Analysis of serum from birds infected with virulent *C. perfringens* isolates identifiedseveralimmunogenic secreted proteins [80]. These proteins included α-toxin, glyceraldehyde-3-phosphate dehydrogenase, pyruvate:ferredoxin oxidoreductase, fructose 1,6-biphosphate aldolase, and a hypothetical protein. These proteins significantly protected broiler chickensagainst a relatively mild challenge, with α-toxin, pyruvate:ferredoxinoxidoreductase and the hypothetical protein offering protection against a more severe challenge [81]. This study provided the first evidence that a subunit vaccine could be useful in controlling necrotic enteritis in chickens. Of interest is that the chicken serum did not react strongly with NetB; possibly because NetB is not highly immunogenic in chickens or the levels of NetB produced *in vivo* are not high enough to promote reliable immune recognition. 

There are no reports in the literature addressing the use of NetB in vaccine formulations, although there are reports of related proteins from other bacteria being successfully used in experimental vaccines. For example, vaccine-based targeting of *S. aureus* α-toxin provided protection against staphylococcal pneumonia [83,84]. In a murine model system, monoclonal antibody-basedtherapy was efficacious for preventionand treatment of this disease [84]. Two distinct anti-α-toxin monoclonal antibodies that blocktoxin activity prevented human lung cell injury *in vitro* and protected experimental animals against lethal *S. aureus* pneumonia. Active immunization with the first 50 amino acids of α-toxin also conferred protection against *S. aureus* pneumonia. This study revealed that both passive and active immunization strategies for prevention or therapy of staphylococcal pneumonia were feasible; similar approaches could be attempted using NetB to protect against necrotic enteritis. A genetically toxoided version of *S. aureus* α-toxin, which was produced by site-directed mutagenesis, has been shown to generate an antigen-specific immunoglobulin G response and to be protective against staphylococcal pneumonia [83]. A similar genetically toxoided version of NetB may have value for necrotic enteritis vaccine design.

## 6. Conclusions

The recently identified pore-forming toxin NetB is a key virulence determinant in *C. perfringens* strains that cause necrotic enteritis in chickens. The dimensions of the pore formed by the toxin have been estimated, but the determination of its exact structure and its comparison to other pore-forming toxins awaits crystallization studies. The toxin is produced by most strains isolated from necrotic lesions, but is less commonly found in *C. perfringens* isolates from healthy birds. As with many of the toxins encoded by *C. perfringens*, NetB production is regulated by the VirSR two-component regulatory system, potentially by a quorum sensing-dependent mechanism. NetB is cytotoxic to chicken LMH cells but not to several other chicken cell lines or Vero cells. Further studies aimed at determining its cellular specificity and identifying its receptor should help to elucidate the precise role that NetB plays in disease pathogenesis. Since NetB is involved in virulence, and is a secreted protein that should be readily accessible to the host immune system, it represents a promising target for vaccine development. Our growing knowledge of the importance and function of NetB may also lead to the development of useful therapeutic agents and biomarkers. The discovery that *netB* is part of a predicted pathogenicity locus that encodes other potential virulence factors indicates that further research is required to further elucidate the mechanism of pathogenesis of this important disease. 
